# Neuromusicology or Musiconeurology? “Omni-art” in Alexander Scriabin as a Fount of Ideas

**DOI:** 10.3389/fpsyg.2016.00364

**Published:** 2016-03-15

**Authors:** Lazaros C. Triarhou

**Affiliations:** Laboratory of Theoretical and Applied Neuroscience and Graduate Program in Neuroscience and Education, University of MacedoniaThessalonica, Greece

**Keywords:** neuroaesthetics, synaesthetics, integrative aesthetics, composers, pianists

## Abstract

Science can uncover neural mechanisms by looking at the work of artists. The ingenuity of a titan of classical music, the Russian composer Alexander Scriabin (1872–1915), in combining all the sensory modalities into a polyphony of aesthetical experience, and his creation of a chord based on fourths rather than the conventional thirds are proposed as putative points of departure for insight, in future studies, into the neural processes that underlie the perception of beauty, individually or universally. Scriabin’s “Omni-art” was a new synthesis of music, philosophy and religion, and a new aesthetic language, a unification of music, vision, olfaction, drama, poetry, dance, image, and conceptualization, all governed by logic, in the quest for the integrative action of the human mind toward a “higher reality” of which music is only a component.

## Introduction: Art to Science

This essay is spurred by the premise that neurobiological mechanisms may be tackled experimentally subsequent to a simple but acuminous behavioral observation, as, e.g., in the case of the discovery of mirror neurons (Rizzolatti and Craighero, 2004) or hippocampal positional cells (Hartley et al., 2014).

Neuroaesthetics attempts to decipher the neural processes that underlie aesthetical experience by focusing on the properties of and interaction among a triad of neural circuits: sensory-motor, emotion-valuation, and meaning-knowledge (Chatterjee and Vartanian, 2014).

York (2010) argues that, if painters are neurologists, as Zeki (2002) implies, then musicians are neurologists who manipulate the auditory brain of audiences for their aesthetic pleasure. Zeki (2002) also suggested that Richard Wagner created his “Tristan chord” being knowledgeable about the operations of the mind and relying on ancient laws of tonality derived from the nature of our perceptive mechanisms, without though having any direct knowledge about brain tissue; through a profound understanding of the workings of the musical brain, Wagner probed it with techniques unique to artists. In that sense, “Wagner was a neurobiologist.”

**FIGURE 1:**
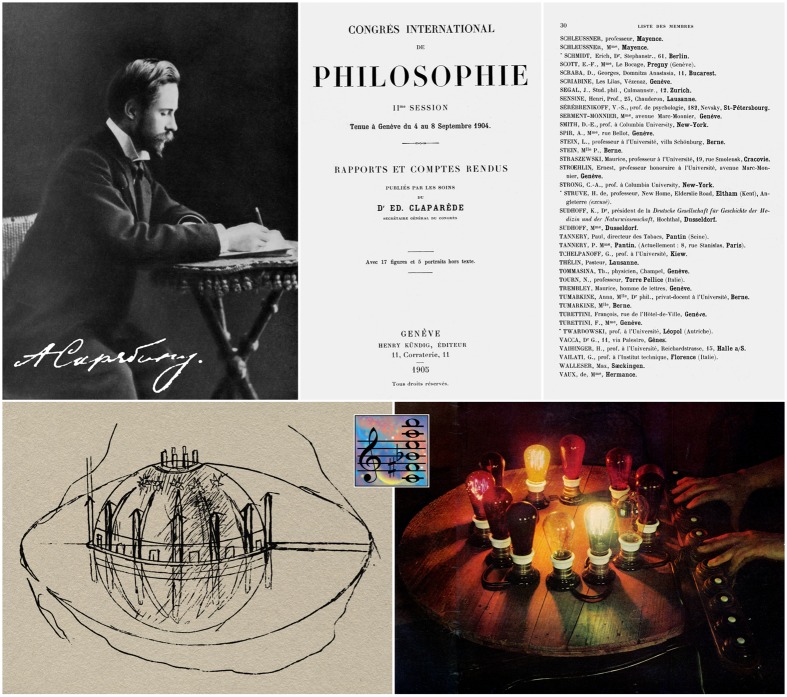
**(Upper left)**: Alexander Scriabin composing (Belsa, 1986); signature etched digitally onto photo from a letter to publisher Mitrofan P. Belaïeff (1836–1904), dated February 11, 1895 (Franke, 1973). **(Upper middle and right)**: Cover of *Reports and Proceedings* of the Second International Congress of Philosophy held in Geneva, and the page showing Scriabin’s name (French spelling) in the roster of members (author’s archive). The Congress was organized by the Swiss neurologist-psychologist Édouard Claparède (1873–1940), nephew of the comparative anatomist René-Édouard Claparède (1832–1871); President of Honor was the theologian-philosopher Ernest Naville (1816–1909). **(Lower left)**: A sketch by Scriabin of the temple where the *Mysterium* was to be celebrated (Scriabine, 1979). It does not bear any inscription, but Scriabin had talked about it to Boris de Schloezer. The edifice, in the shape of a semi-dome, would be elevated over the water plane in the middle of a lake, such that, by its reflection, it would appear as a perfect sphere; color shafts of light would give the impression of a varying architecture. There are six portals (12 in total), and a crown of stars near the vertex. The meaning and function of the “pillars” is elusive. **(Lower right)**: The color keyboard, executed by the physicist Alexander Moser upon Scriabin’s commission, and designed and constructed specially for the performance of *Prometheus*, the first score to include a color instrument. It is a wooden circle of 12 lamps: seven lamps according to the scale of the spectrum (red, orange, yellow, green, sky-blue, blue, violet) and five additional lamps linking the extreme colors of the spectrum and forming a transition from violet to red, rosy, rosy-red, etc. This circle corresponds to the circle of fifths in music, the red standing for C, the orange for G, the yellow for D, and so forth (Hope, 1970). The instrument is housed in the Scriabin Memorial Museum in Moscow. **(Inset, center)**: Scriabin’s “mystic chord” in fourths.

The present discussion centers around the avant-garde composer and pianist, Alexander N. Scriabin (1872–1915), an isolated phenomenon in Russian music (**Figure 1**), who erected classical edifices of sound, independent, perfectly finished (Altschuler, 1972; de Schloezer, 1987; Triarhou, 2015). He is reckoned one of music’s revolutionaries, his compositions being genial, eloquent, articulate, impassioned (Mann, 1980). Every piece of his is a multifaceted crystal in which the powerful breath of nature and the intense contemplation and imagination of the artist merge into one (Shaborkina, 1980).

In his late works Scriabin searched for a new musical language, abandoned tonality, experimented with colors, and delved into innovations of art, scenery, lighting, plastic movement, paths that logically anticipate the experimentation of contemporary artists and the multimedia aspects fashionable today (Cohn, 1968). Many of these ideas have become standard on our horizon, yet they were discernible in 1910 in the musical cosmos of Scriabin (Scriabine, 1987).

Pianist Igor Zhukov, a deeply versed Scriabinian, explains that, in his majestic temple, Scriabin followed no one and left no one to follow him (Shukow, 1999); in the brief 25 years of his creative life he “penetrated distant worlds and touched upon cosmic mysteries.” Scriabin meant “Creation” as a triad comprising unremitting desire, unlimited enthusiasm, and a purpose or determination that the individual must continually advance and enrich. The titanic workings of Scriabin’s thought attest to him being no out-of-touch philosopher (Shukow, 1999). Scriabin’s attempt to embrace all branches of knowledge led him to ponder into psychology, philosophical discourse and the ambience of Hindu culture (Bowers, 1972). He became a member of the Moscow Philosophical Society and attended its meetings regularly (de Schloezer, 1987). During the summer of 1904, he studied Wundt’s psychology (Scriabine, 1979). In September 1904 he attended the Second International Congress of Philosophy (**Figure 1**), which featured speakers the caliber of Henri Bergson, Pierre Boutroux, Wincenty Lutosławski, and Gaston Milhaud (Claparède, 1905).

## Integrative Aesthetics: Return to Omni-Art

As he dabbled into philosophy, Scriabin became interested in the relationship of sound and tonality to color; his musical language entered a new phase and led to his symphonic works *Le poème de l’extase* and *Le poème du feu* (Sabaneiev, 1974). In his words, “Through music and color, with the aid of perfume, the human mind can be lifted outside or above merely physical sensations into the region of purely abstract ecstasy and intellectual speculation.” In his speculations, Scriabin relied wholly on intuition, science as yet having uttered no word (Runciman, 1915). He was defined as a solipsist, adhering to the philosophical stance that only knowledge of self is possible, and for each individual, mind is the only thing sure to exist (Bowers, 1972).

Scriabin was convinced that the experience of colors would enhance the experience of sound, and suggested that audiences would absorb his *Prometheus* more fully if they were bathed in colored light corresponding to the musical flow. In Scriabin the use of abstract colors over the dimension of time, not developed for the cinema yet, may be technologically primitive, but the music is exceedingly sophisticated (Macdonald, 1978).

*Prométhée, Le poème du feu*, op. 60 (1909–1910) is his last completed symphonic work. It is a hybrid between a symphonic poem, a concealed piano concerto, a sonata augmented by an introduction and coda, and a multimedia cantata for four-part wordless chorus and full orchestra amplified by extra strings and winds, organ, bells, glockenspiel, celesta, two harps, and a keyboard called *Svetovaya klaviatura* or *Tastiera per luce*, which makes no sound but produces colors (**Figure 1**). Opus 60 was Scriabin’s first systematic attempt at connecting music with colors. The use of light is indispensable and complementary to the sound in one art form, such that listeners would discern the higher-level harmonic content of the music and comprehend the Symbolist narrative of the drama that Scriabin intended his symphonic poem to embody (Gawboy, 2010).

One reason for including light elements in the *Poem of Fire* is obviously fire, which occupies a central position in Russian culture both for its comforting value in cold winters and for its destructive force. Scriabin studied and prized Aeschylus’ *Promethean Trilogy* and Dante’s *Divina Commedia*, and kept Sophocles’ *Tragedies* and Plato’s *Symposium* in his library (Bowers, 1969; de Schloezer, 1987). He interpreted *Prometheus* as the disobedient venturer who, besides bearing fire to humans, also presented them with the fire of intelligence, enabling them to be superior to God (Bowers, 1972). Scriabin had absolute faith in the unlimited power of human free will (de Schloezer, 1987). One of his philosophical tenets was that “A God who needs adoration is no God” (Scriabine, 1979). He uttered that the artist is more important than God, and that politicians and bureaucrats should not be praised: “Writers, composers, authors and sculptors are the first-ranking in the Universe, first to expound principles and doctrines and to solve world-problems. Real progress rests on artists alone. They must not give place to others of lower aims” (Bowers, 1972; Garcia, 2004).

Scriabin used a color scale founded on the piano-tuner’s “cycle of fifths,” and wrote music in a novel harmonic system. He placed 12 hues on his 12-note keyboard, with colors following more or less closely the bass notes of his harmonies (Eaglefield Hull, 1916).

Scriabin’s death prevented the completion of his conceived masterpiece, *Mysterium*, which would present a theosophical interpretation of the evolutionary psychology of humanity, right up to its purifying ecstasy and cosmic freedom (Rüger, 1980; de Schloezer, 1987). *Mysterium* was the dream of unifying humanity in a common beatitude of ecstasy which would transfigure and deify humans and the Universe and would lead to a new Cosmos (de Schloezer, 1987). By *Mystery* Scriabin did not mean anything baﬄing or misunderstood; he meant it in the sense of Dionysian and Eleusinian mysteries, and also in the Christian sense, enacted in temples and cathedrals (Bowers, 1972). It was to be a liturgical drama which would effect a synthesis of all arts (de Schloezer, 1987).

*Mysterium* would for the first time reveal a “total art,” a new aesthetic language including what Scriabin felt was most likely a unification of music, colored lights, mist, incense, fragrance, drama, poetry, dance, in his quest for an integration of all the senses. It would be performed against the backdrop of the Himalayas (**Figure 1**) as an “immense liturgical rite” over seven days and seven nights and elevating humankind to a higher world (Witztum and Lerner, 2014). Spectators would be participants in an “oneness” of performers and audience.

Scriabin adored chaotic fortissimi in his crashing finali, blending everything from “trumpets of Archangels to vortical dances spinning in disequilibrium, as if a thousand bells had gone wild, a holy last dance that accomplishes all, before the final instant of dematerialization” (Bowers, 1969). In 1912 he had announced that he was composing *Icarus*, a symphonic picture in which he would depict the flight and fall of the tragic hero of Greek mythology; he planned to incorporate into the orchestra the sound of an aeroplane propeller to create the illusion of fluttering wings (Bowers, 1969; Levi, 2000).

The roots of many Russian avant-garde composers and early Futurists lay in the music of Scriabin. Futurism in music remained a potent and central force in Russia for a much longer period of time compared to Western Europe, one of the reasons being that it had attracted a much wider range of composers. Although Scriabin can hardly be deemed a true Futurist, he provided the stimulus for others who followed (Levi, 2000).

In the final phase of his life, Scriabin studied Sanskrit and took yoga exercises. What animates the artistic design of *Mysterium* is both the synthesis of all arts and the inclusion of elements beyond aesthetics proper (de Schloezer, 1987). The idea of a unity of all arts, the basis of the doctrine of an “Omni-art,” arose from Scriabin’s intuitive experience as, to him, tones had no separate existence from colors, images or concepts.

Scriabin’s work is one of the most original of all music, of no less caliber than that of Schoenberg, Bartók, Prokofiev, or Stravinsky. Scriabin put to music questions that had not been put before him. Creator at the jostling transition of worlds between the 19th and 20th centuries, divided between romanticism, and modernism, Scriabin made of art a sort of religion and initiation appealing to transform life (Clément, 2015).

Mixed media is not a modern invention. Odorama, smellavision, son-et-lumière, and laser-light shows all have their historical place (Cytowic, 2002). When *son-et-lumières* are combined, the modern artist that comes to mind is the French composer Jean-Michel Jarre, known for his spectacles of music with laser displays and fireworks (Duguay, 2011).

Did Scriabin involuntarily see colors when he heard musical pitches or did he intentionally relate certain pitches to specific colors? Harrison (2001) comments that “there is considerable doubt about the legitimacy of claims made on Scriabin’s behalf.” Galeyev and Vanechkina (2001) conclude that “the nature of Scriabin’s color-tonal analogies was associative; accordingly, the existing belief that Scriabin was a distinctive, unique synaesthete is placed in doubt.” Oliver Sacks (2008) upheld the thesis that Scriabin’s tone-color associations “may have represented a conscious symbolism rather than actual synaesthesia.”

Part of the perplexity of the problem could pertain to the common etymological root of the words *aisthēsis* (sense and sensation as in physiological psychology) and *aisthētikē* (aesthetics as in philosophy) from the Greek verb *aisthanesthai* (to feel).

The Austrian visual artist Johannes Deutsch (2012) differentiates between synaesthesia as a neurological condition of exceptional sensory perception and a long-standing tradition of “synaesthetic oeuvres” in the history and theory of art and culture; the idea then emerges that Scriabin linked musical tones to colors in *Prometheus* to consciously generate a “syn-aesthetic symphony.”

An early pioneer who had combined art and science in the quest for a synthesis of rational analysis and imagination was the Italian physiologist and painter Filippo Lussana (1820–1897). Influenced by phrenology and Newtonian physics, Lussana formulated, between 1865 and 1873, a theory of colored-hearing synaesthesia; he suggested the existence of a “center of chromatic talent” in the inferior frontal gyrus in association with the language areas, conferring an emotional sense to color and sound, and elaborated a scale to correlate color and note vibrations (Lorusso and Porro, 2010).

**FIGURE 2:**
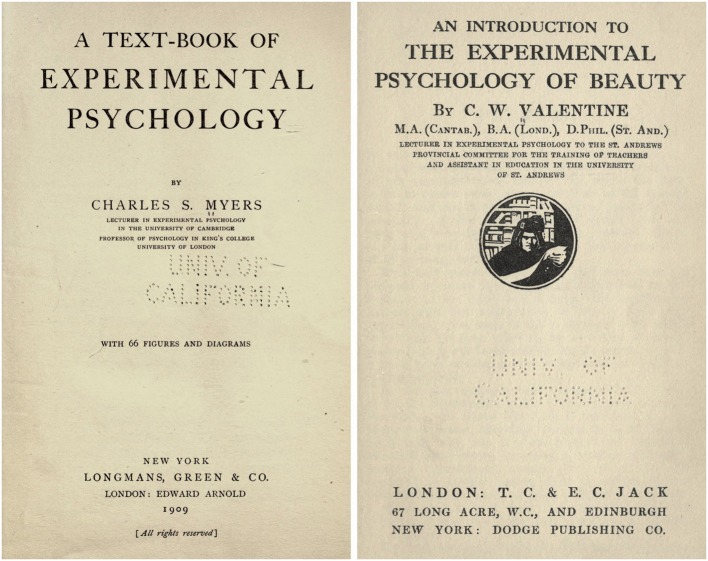
**Title pages of the psychology textbooks by Myers (1909) and Valentine (1913).** Credit: The Open Archive (www.archive.com).

Scriabin’s conception of multimodal aesthetics receives support from an interview granted by the composer to physician and psychologist Charles S. Myers (1873–1946) at the University of Cambridge (Myers, 1914; Peacock, 1985), in the presence, most likely, of educational psychologist Charles W. Valentine (1879–1964), who had just published a book on the psychology of beauty (Valentine, 1913). Myers (1909), a talented violinist, holds a pre-eminent position in the history of British psychology. Myers (1909) became the first physician whose whole duty was to teach experimental psychology and published his pioneering *Textbook* (**Figure 2**). Myers (1909) writes that Scriabin’s “chromaesthesia” and simultaneous presentation of the appropriate color to the eyes enhanced the musical effect. In true synaesthetes, a specific tone always induces a particular color, involuntarily and persistently. In Scriabin it was the refinement of his intellect and skill that led him to develop such connections.

At the dawn of history, humanity knew only one inchoate “Omni-art,” in which the elements of dance, music, painting formed a perplexed tissue of motoric, auditory, and visual sensations; Scriabin thought that a memory of this ancient period had survived in Classical Greek theater, where all “heterogeneous” components were closely interrelated (de Schloezer, 1987). After the Renaissance, the various branches of the arts became autonomous, reaching startling perfection in music, literature and painting independently (Sabaneiev, 1974). At the turn of the 20th century, right after the advent of electricity, the arts of movement, the play of light, and the symphony of colors began to develop and reunite. Wagner formulated such ideas vaguely, but it was Scriabin who expressed them much more clearly: “All arts must be united in one work whose exaltation will be followed by an authentic vision of a Higher Reality.”

## Chord of Fourths: Universal Aesthetic Object?

Scriabin’s complex harmonic theory was systematic and disciplined. Departing from the major-minor context of classical harmony, he invented something new and unique, “a total entity within itself,” leveling the vertical and horizontal differences between harmony and melody to a single unit (Mackey, 1994). Scriabin considered “melody to be harmony in horizontal form and harmony to be melody in vertical form … Melody is harmony unfurled, harmony is furled melody” (Bowers, 1969).

The later works of the composer reveal an outright disregard for traditional music theory (Gunst, 1915). He created his new chord by selecting his preferred sounds from Nature’s harmonic chord and building them up by fourths. The result is a chord of interest and beauty.

The “central” Scriabinian sound (“We face an ecumenical consciousness that experiences a plethora of conscious states vertically in time and horizontally in space”) developed from a layering of thirds on the dominant to a stack of fourths. The six-note chord, which opens *Prometheus* as a symbol of the all-embracing primordial chaos, the “Promethean chord,” is structured in augmented, pure and diminished fourths: A – D-sharp – G – C-sharp – F-sharp – B (colors: blue-violet-gray). A “mystic chord” of fourths (C – F-sharp – B-flat – E – A – D) also appears in the middle of the *Fifth Sonata* for piano.

Newer research suggests that the matching of color with the “mystic chord” in *Prometheus* originates as a compositional device for the purpose of opening up the possibility for a synaesthetical experience (Secor, 2013). Harmonics are based on a six-note scale representing the seventh, eighth, ninth, tenth, twelfth, and thirteenth overtones of a fundamental yielding, in the scale of C, the tones C – D – E – F-sharp – A – B-flat. A series of such tones, e.g., C^1^ – F^1^-sharp – B^1^-flat – E^2^ – A^2^ – D^3^, simultaneously played in true, untempered intonation, produces what he terms a “single sound.” The “mystic chord” (**Figure 1**), with its arrangement in fourths, rather than thirds, and its transpositions and contrapuntal elements, led to the door of atonality and polytonality, prefiguring Schoenberg’s dodecaphony.

## Cerebral Nuances

Novel constructs by composers, in the quest for aesthetic verity, may offer tips to neuroscientists in the exploration of brain mechanisms that mediate aesthetic perception. For example, questions may be posed or hypotheses formulated, in the experimental setting, with regard to how universal is the aesthetic perception of Scriabin’s chord of fourths compared to the classical chord of thirds? Or how do sound, light, odor, and touch engage in tandem to produce a multisensory experience in the logic of the human brain, and what neural circuits underpin such an integrative sense of beauty? Are such occurrences commonalities or differentiations among individuals, based on cultural prehistory, musical training, exposure to the visual arts, among other variables?

Syn-aesthetics could provide a window into the neural correlates of cross-modal associations, and the mind of a composer could conceivably open up windows to probe into brain function. There is a characteristic, independent of learning, or cultural ethos, to all that is experienced as “beautiful,” common to all and peculiar to none; it lies in a simple neurobiological fact, that whenever an individual experiences beauty, whether the source is visual, musical, moral, or mathematical, there is a correlate in the form of metabolic activity in an anatomical component of the emotional brain, namely, field A1 of the medial orbitofrontal cortex (Zeki, 2014). The medial orbitofrontal cortex and the adjacent cingulate cortex respond to various sources of pleasure, including music (Ishizu and Zeki, 2011), even architectural space (Vartanian et al., 2013). The study of performing arts, such as dance, suggests a possible role of visual and sensorimotor areas in an automatic aesthetic response, particularly a system comprising mirror neuron areas in the premotor and parietal cortices for perceiving and executing actions, as well as superior temporal sulcus and occipital cortical areas (Calvo-Merino et al., 2008).

Artists are driven by an urge for impact. Science can look at their work to find a “naïve physics” that uncovers deep and olden insights into the workings of the mind; the discrepancies between the real world and the world depicted by artists may reveal as much about the brain within us as the artist reveals about the world around us (Cavanagh, 2005).

In musicians, mirror neurons combine auditory and visual (action-observation) systems and may instigate a bidirectional interaction between players and audience. Performers feel the emotional essence of the music and communicate it to listeners; audiences do not merely observe or hear a performer, but perceive emotional content (Molnar-Szakacs and Overy, 2006; Svard, 2010). The reciprocal may also be happening: the affective state of the audience, individually, or collectively, may mirror on soloist, conductor, orchestra. Musicians have known all along that each performance of the same score is particular and unique.

Scriabin sought his formation in a synthesis of the arts, interlaced with colors, poetic elements, perfumes, voluptuous flower scents, and tactile association (caresses) between audience and performers: “The creator, exerting his influence on listeners, supposing the action is musical in nature, is reciprocally influenced by them” (de Schloezer, 1987).

The composer incarnated the will to transform the world through the power of a total art, his new synthesis of music, philosophy, and religion (Gorenstein, 1992). Scriabin saw Omni-art as a counterpoint or polyphony of individual arts. In the “contrapuntal” *Mysterium*, he included the so-called lower senses, touch, taste, and olfaction, albeit still governed by logic. Those elements did not have to occur simultaneously, but, like in a fugue, their continuity could be disrupted and restored at will. The return to the primordial state of an integrative art would not be merely a recapitulation, but rather, a transfiguration toward universal beauty (de Schloezer, 1987).

Scriabin called the idea of an intimate intermingling of all artistic and philosophical disciplines, and their essential oneness, a “counterpoint,” as opposed to Wagner’s parallelism of music and drama. de Schloezer (1987), in his account of the life of Alexander Nikolaevich, noted: “To Scriabin, Ultimate Reality was Omni-art, of which music is only a component.” Decades later, the cell biologist de Duve (2002), the discoverer of the lysosome, would conclude his own account of the saga of life on a concordant note: “I have also vibrated in different registers, in resonance with poets, writers, artists, and musicians who moved me by their works and performances. On exceptional occasions, I have felt close to something ineffable, utterly mysterious but real, at least to me, an entity that, for want of a better term, I call Ultimate Reality.”

## Author Contribution

LCT conceived the idea of the paper and wrote it in its entirety.

### Conflict of Interest Statement

The author declares that the research was conducted in the absence of any commercial or financial relationships that could be construed as a potential conflict of interest.
